# Clouded leopards, the secretive top-carnivore of South-East Asian rainforests: their distribution, status and conservation needs in Sabah, Malaysia

**DOI:** 10.1186/1472-6785-6-16

**Published:** 2006-11-08

**Authors:** Andreas Wilting, Frauke Fischer, Soffian Abu Bakar, K Eduard Linsenmair

**Affiliations:** 1 Theodor-Boveri Institute of Animal Ecology and Tropical Biology, Institute of Biosciences, University of Würzburg, 97074 Würzburg, Germany; 2 Sabah Wildlife Department, Blok B, Wisma Muis, 88100 Kota Kinabalu, Sabah, Malaysia

## Abstract

**Background:**

The continued depletion of tropical rainforests and fragmentation of natural habitats has led to significant ecological changes which place most top carnivores under heavy pressure. Various methods have been used to determine the status of top carnivore populations in rainforest habitats, most of which are costly in terms of equipment and time. In this study we utilized, for the first time, a rigorous track classification method to estimate population size and density of clouded leopards (*Neofelis nebulosa*) in Tabin Wildlife Reserve in north-eastern Borneo (Sabah).

Additionally, we extrapolated our local-scale results to the regional landscape level to estimate clouded leopard population size and density in all of Sabah's reserves, taking into account the reserves' conservation status (totally protected or commercial forest reserves), their size and presence or absence of clouded leopards.

**Results:**

The population size in the 56 km^2 ^research area was estimated to be five individuals, based on a capture-recapture analysis of four confirmed animals differentiated by their tracks. Extrapolation of these results led to density estimates of nine per 100 km^2 ^in Tabin Wildlife Reserve. The true density most likely lies between our approximately 95 % confidence interval of eight to 17 individuals per 100 km^2^.

**Conclusion:**

We demonstrate that previous density estimates of 25 animals/100 km^2 ^most likely overestimated the true density. Applying the 95% confidence interval we calculated in total a very rough number of 1500–3200 clouded leopards to be present in Sabah. However, only 275–585 of these animals inhabit the four totally protected reserves that are large enough to hold a long-term viable population of > 50 individuals.

## Background

Many large carnivores are under severe threat from permanent loss of suitable habitat as well as direct persecution, making their protection a top conservation priority. Due to their spatial requirements, these species are among the first to suffer when human populations expand and alter pristine habitats [1]. The conservation of large predators is vital, due to the critical role they play in the long term persistence of biodiversity and stabilization of ecosystems [2-7]. Detailed management plans are a prerequisite for the effective conservation of such keystone species. These plans need to be based on sound, reliable and accurate information regarding the ecology of the species as well as the status and health of the population. Hence, reliable methods providing accurate data on abundance, population trends, and threats are of extreme importance. However, for many carnivores, basic information on life history parameters and population ecology is lacking. This shortcoming is a significant challenge for wildlife managers [8]. In the recent past, most studies on large carnivores applied radio telemetry and camera trapping to estimate home range sizes and densities. [9,10] These methods, however, are very costly, time consuming, labour intensive and require experienced investigators.

The value of simpler methods such as track counts is often underestimated, even though recent studies have shown the power of such techniques [11,12]. This study applied a technique described by Smallwood and Fitzhugh [11] to identify individual mountain lions (*Puma concolor*) by their tracks, allowing for inventorying and monitoring carnivores at low costs with relatively little effort [13].

The clouded leopard is the largest cat of Borneo, weighing between 11–25 kg. The name "clouded leopard" refers to the distinctive cloud-like fur pattern [14]. Several adaptations such as the proportionately short legs, the large, broad paws with sharp claws and the exceptionally long tail give clouded leopards amazing arboreal skills, including the ability to climb down trees head-first [15].

The clouded leopard currently shows a rather patchy distribution throughout its range [16]. Many of the remaining forest areas are too small to ensure the long-term persistence of clouded leopard populations making effective monitoring and management measures necessary. Their partly nocturnal and far-ranging behaviour [9], their low densities [17], and the fact they inhabit densely vegetated habitats and remote areas makes the censusing and monitoring of clouded leopards extremely difficult. Consequently, little is known about their behaviour and status. A total of only seven clouded leopards have ever been radio collared [9,17,18] and the study of Grassman et al. [9] is the only one which systematically surveyed a clouded leopard population *in situ*. Most other available information on clouded leopard ecology is anecdotal [19,20], based on local interviews [21,22] and a few sighting reports [23,24], or stems from captive animals [15,25]. According to Davies and Payne [26] and Rabinowitz et al. [21], clouded leopards can regularly be seen walking along jungle trails and roads in Borneo, making previous assumptions of them being strictly arboreal by Pocock [20] and Wood [27] questionable.

At present, the clouded leopard is listed by IUCN as vulnerable [28] and included in CITES Appendix I. Based on interviews in Malaysian Borneo in 1986, Rabinowitz [21] concluded that clouded leopards seemed not to be in imminent danger of extinction there. However, because of ongoing deforestation and logging this may have changed.

The goal of this study was to determine the local population density of clouded leopards in Tabin Wildlife Reserve. A specific objective was to develop a reliable and cost-effective monitoring technique that could be widely transferred to other protected areas. In this paper we present the first study that combines a rigorous track classification method with a capture-recapture model for research on an elusive carnivore. Furthermore we extrapolated the calculated density of clouded leopards to a landscape level to draw inferences on their distribution region-wide.

The ultimate goals of the study were to contribute to the knowledge of the ecology of clouded leopards and to give the first status assessment for Sabah of one of the most threatened cat species in Asia.

## Results

### Tracking

**Figure 1:**
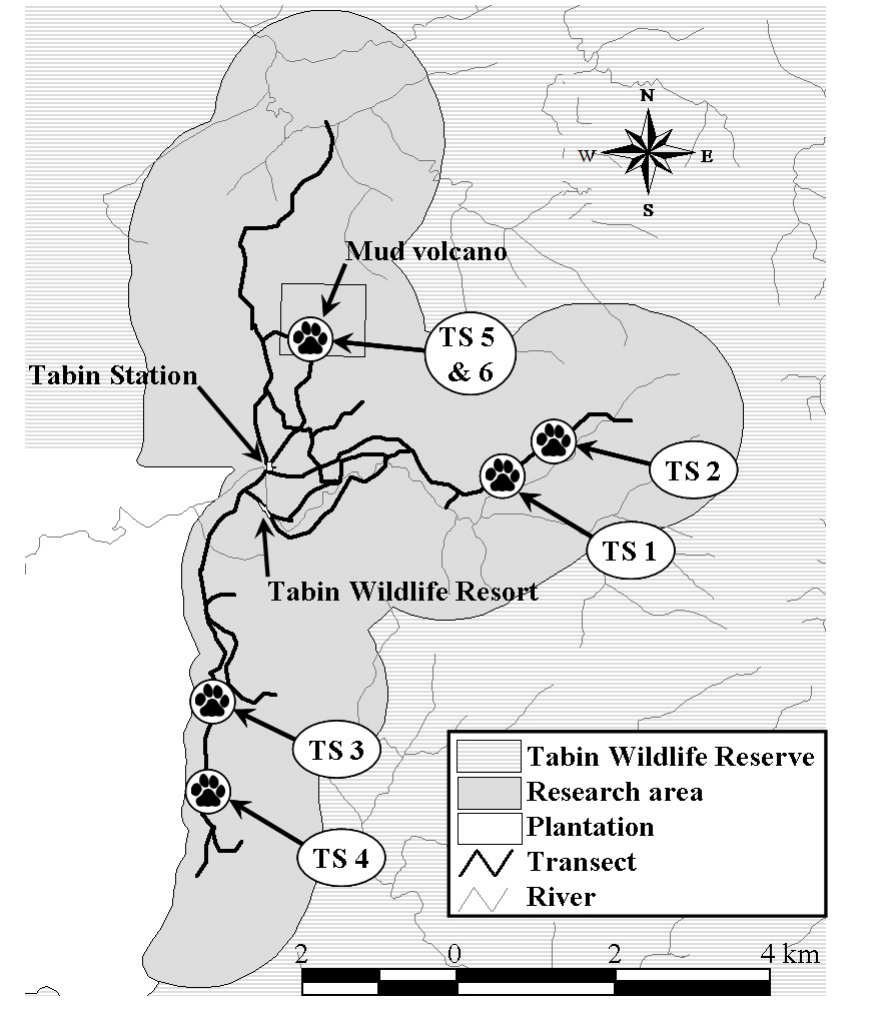
Location of the track sets (TS) of clouded leopards within the study site.

During the field work six track sets consisting of 1, 4, 8, 10, 13 and 14 pugmarks were recorded. All tracks were in good condition and were measured several times. Track set (TS) 3 consisted of only one mark but was found after a sighting of an adult male and could therefore be assigned to this individual. TS 1 and TS 4 were smaller in size. The last three TSs (TS 2, 5, 6) were larger and similar to the one track found after the above-mentioned sighting. The left front pugmarks of these TSs were always smaller in size than the right front tracks of the same TSs. Two of those three TSs, TS 5 and TS 6, were recorded on a mud-volcano (Fig. 1). This mud-volcano is visited on a regular basis by many animals as a natural salt lick. TS 3 and TS 4 were recorded along the southbound road, whereas TS 1 and TS 2 were found along the old logging road running east to west. Although two transects followed existing jungle trails, which were supposed to be used by clouded leopards frequently, no tracks were spotted there. This might have been as a result of leaves covering most of the ground. In addition to tracks observed on the roads, eight TSs were detected along stream transects. These tracks were of poor quality and could neither be allocated without doubt to clouded leopards nor could be measured accurately. Thus these tracks had to be excluded from the analysis.

### Individual identification by tracks

A paired t-test indicated that the means of the total width from each TS from front left and right tracks differed significantly (*n *= 5, *t *= -3.3, *p *= 0.03). Although all other track characteristics appeared to be similar for respective left and right measures (all p < 0.05), we analyzed front left and right marks independently to account for the differences in track sizes. In contrast, means from rear left and rear right tracks of the same clouded leopard were not statistically different (*n *= 4, *t *= -0.5, *p *= 0.65). Thus we could combine left and right rear tracks for the analysis to increase the level of statistical power. Due to the small sample size, tracks found on slightly different substrates were combined for analysis, but substrates differed more within one track set than between the track sets.

In our analysis in each of the three cases (independent analysis for rear, front left and front right tracks) the first two principal components (PC) explained over 97 % (eigenvalues for rear tracks × 1 = 13.4, × 2 = 0.3; for front left tracks × 1 = 13.5, × 2 = 0.3; for front right tracks × 1 = 13.8, × 2 = 0.1) of the total information within all 14 variables with PC 1 explaining always over 96 %. This finding suggested that the first PC would already be sufficient to differentiate the tracks, but a two-dimensional graph with the first two PC would be preferable for a better illustration.

**Figure 2:**
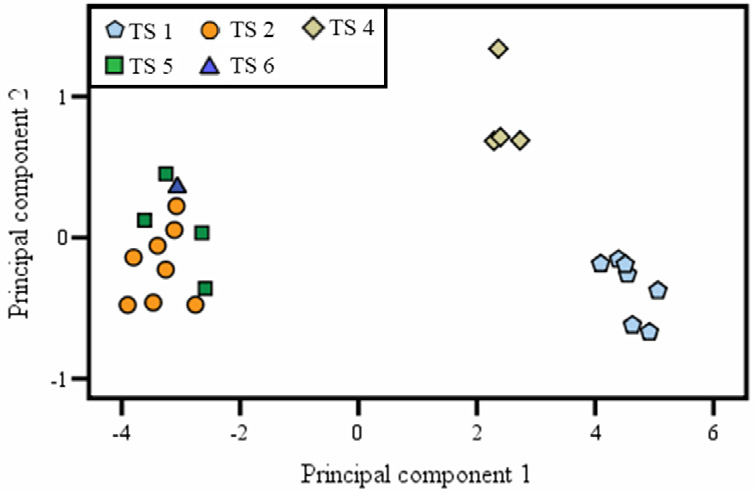
**Principal component loadings of clouded leopard (*Neofelis nebulosa*) rear tracks**. Tracks derived from left and right rear pugmarks were taken in Tabin Wildlife Reserve, Sabah, Malaysia. Different symbols indicate tracks belonging to the same track set (TS).

**Figure 3:**
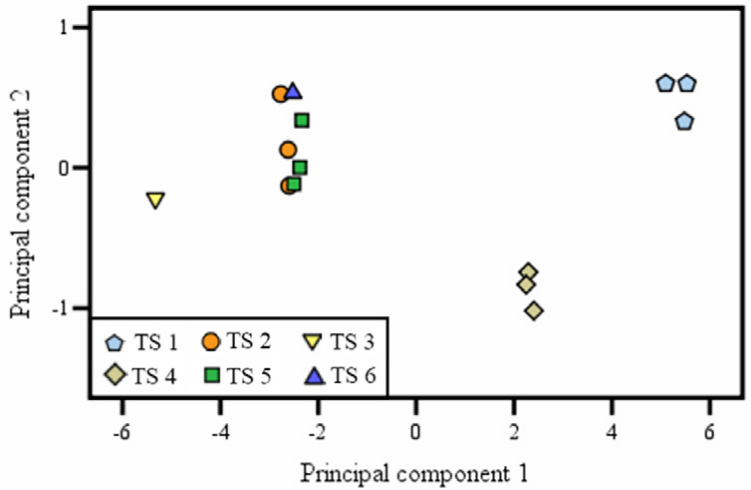
**Principal component loadings of clouded leopard (*Neofelis nebulosa*) front left tracks**. Track derived from front left pugmarks were taken in Tabin Wildlife Reserve, Sabah, Malaysia. Different symbols indicate tracks belonging to the same track set (TS).

Figure 2 shows the scatter plot for the rear tracks. All tracks from both TS 1 and TS 4 form clusters, but are spatially separated in the scatter plots, suggesting that these track sets were left by two different clouded leopards. The tracks of TS 2, TS 5 and TS 6 intersected with each other and grouped together in space suggesting that those tracks sets, found at different locations and dates, were of the same individual. Since TS 3 consists of just one pugmark it is only found on figure 3, which shows the principal component analysis (PCA) of left front marks. The track from TS 3 is spatially separated in the scatter plot suggesting that this track was produced by a different clouded leopard. In summary, the track sets were probably formed by four different clouded leopards.

### Population size and density

The track classification technique resulted in a calculated minimum number of four clouded leopards present in the surveyed area between March and August 2005. The results of the closure test (tracks: *z *= -0.118, *p *= 0.453) provided no evidence of violation of the closure assumption (Table 1). The model selection algorithm of capture identified M_0 _as the most appropriate model for both analyses. We adhered to this suggestion and present population estimates under the M_0 _model (see discussion). The analysis determined average capture probabilities to be 0.06 and the estimated probability that a clouded leopard was captured at least once was 0.80 (Table 1). We estimated five (± 2.26 SE) clouded leopards to be present in the research area on the basis of a capture-recapture analysis of the tracks. To estimate the true density we calculated an approximately 95% confidence interval. We used a range of 2 SE on the upper side of the point estimates, but we did not use 2 SE on the lower side of the point estimate, because a calculated number below the threshold of identified individuals would not be reasonable. Thus we set the number of differentiated individuals as the lower limit. We did not use the confidence interval calculated by CAPTURE, because the guidelines of CAPTURE noted that low capture probabilities will lead to extremely wide confidence intervals that hold little information on the true population size [29].

In order to calculate the population density within the reserves, the effective surveyed area also had to be estimated. Applying equation 1 and data from Grassman [9], a buffer width (*W*) of 1.58 km was generated. Buffering all transects resulted in the research area (*A*), which comprised about 56 km^2 ^(Fig. 1).

We estimated a rough density of nine individuals per 100 km^2 ^in Tabin Wildlife Reserve. The true density lies most likely between our approximately 95 % confidence interval of eight to 17 individuals per 100 km^2^.

### Management implications

**Figure 4:**
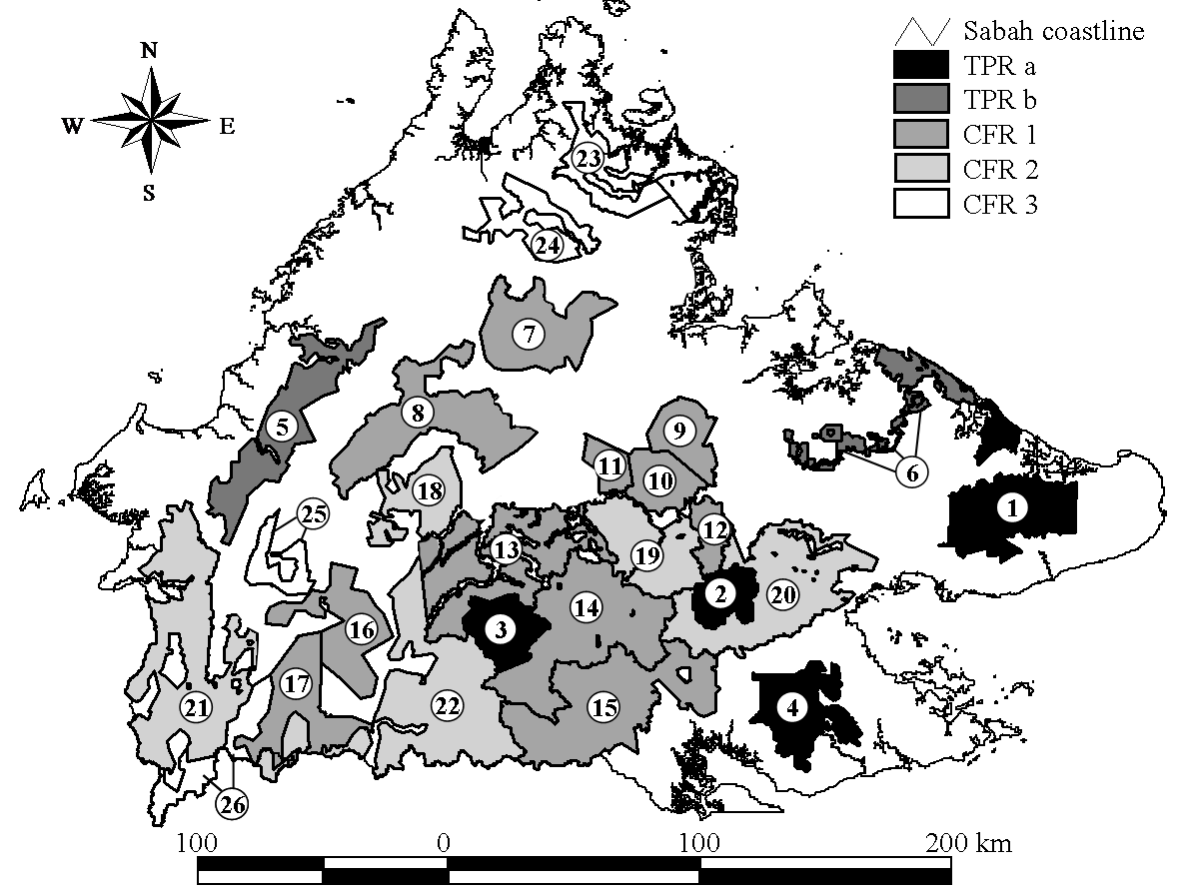
**Protected areas in Sabah, ranked according to their protection status and importance for clouded leopard conservation**. Names of reserves are shown in Table 1. TPR a = Totally protected reserve with a stable clouded leopard population, TPR b = Fragmented TPR, CFR 1 = Commercial forest reserve with clouded leopards present, CFR 2 = CFR with unknown clouded leopard status, CFR 3 = CFR with clouded leopards absent.

Based on the very rough density of nine individuals per 100 km^2^, we assume that at least all areas smaller than 350 km^2 ^might be too small to contain a stable population of clouded leopards (> 50 individuals). Thus, only protected reserves larger than 350 km^2 ^as well as reserves connected to others can be considered as potential clouded leopard refuges. Table 2 shows the list and status of all these protected areas in Sabah. In total they comprise an area of about 30 000 km^2^, about 41 % of Sabah's land surface. The presence of clouded leopards is confirmed in approximately 25 % of Sabah based on the last faunal survey and direct observations (Fig. 4). About 12 % of Sabah's forest reserves were not included in the last faunal survey, thus no information about the clouded leopard's status in these areas is available. Taking this into account, the potential distribution of clouded leopards is about 37 % of Sabah. Only six reserves are totally protected (Table 2), covering an area of only 7 % of Sabah. One of these reserves, Crocker Range NP, is divided by a mountain range with elevations higher than 1500 m. Based on information of previous faunal surveys, clouded leopards in Borneo only populate areas below 1300 m [26]. We believe that the areas in Crocker Range NP below 1300 m are too small and too fragmented to sustain a viable clouded leopard population. We completely excluded Kinabalu Park as a potential refuge for the same reasons. The Kinabatangan Wildlife Sanctuary consists of small forest fragments too small and isolated to sustain a viable clouded leopard population. Therefore Kinabatangan Wildlife Sanctuary and Crocker Range NP were only classified as totally protected reserve (TPR) b in figure 4. The last remaining four refuges (TPR a in figure 4), covering only 5 % of Sabah, are isolated from each other with only one connection via commercial forest reserves between Maliau Basin and Danum Valley. Therefore, most of the potential distribution range of clouded leopards is located in commercial forest reserves, where selective logging and licensed hunting is permitted.

In total, we estimated a very rough number of 1500–3200 clouded leopards inhabiting reserves in Sabah, based on the density of eight to 17 individuals per 100 km^2 ^(see discussion). Areas where no data about the presence of clouded leopards were available or reserves which are smaller than 350 km^2 ^and not connected to other reserves were excluded. According to our analysis, the four remaining reserves of total protection in Sabah harbour less than 20% of the entire clouded leopard population.

## Discussion

Davies and Payne [26] provided the only previous, but very rough, density estimate of clouded leopards in Sabah. They assumed that 12 one-square kilometre study areas were surveyed accurately enough to detect clouded leopards. On the basis of three observations (tracks or sightings), they concluded a density of one individual/4 km^2 ^or 25 animals/100 km^2^. This estimation was intended as a base for further research, but became a "quoted fact" in literature [30].

Our results lead to the assumption that Davies' and Payne's [26] approach most likely overestimated the true density even though we can not prove that Tabin's clouded leopard population is representative for other areas in Sabah. The expected relatively large and overlapping homeranges of clouded leopards might be just one of the reasons why Davies' and Payne's method is not accurate. Our density estimate incorporates a few inaccuracies as well, mainly due to the limited number of good track sets which could be obtained in the relatively short study period. We chose the null model M_0 _with constant capture frequencies following Trolle and Kéry [31] in their study on ocelots, although we are aware that due to the small sample sizes, selection criteria were defaulted to the null model M_0 _with minimum parameters involved. We are also conscious that the M_0 _is sensitive to violations of the underlying model assumption of homogeneous capture probabilities [29,32], leading to underestimates of the true density and of the standard error. In contrast to the M_0 _model, the Jackknife population estimator M_h _incorporates variable capture probabilities of individuals [29,32]. Karanth and Nichols [10,33,34] pointed out that this model tends to be the most robust to deviations from model assumptions. However the Jackknife estimator does not provide an adequate estimation of population sizes if only a few animals are recaptured [29]. Based on our experience in the research area, the Jackknife estimator tends to overestimate the true density. We are more concerned about overestimating the true density, since this will automatically lead to an underestimation of the risk the population faces [35]. Even though the Chao M_h _estimator is more robust against low capture probabilities compared to Jackknife it could not be applied, because the capture frequencies did not satisfy the conditions asked for. In addition, we believe that due to the social structure of clouded leopards and the non-invasive nature of track sampling, the encounter probabilities are almost homogenous, in contrast to the use of camera-trapping where the animals' behaviour might lead to unequal access to the camera-traps [10,33,34,36]. We would like to emphasize that our estimates of nine individuals per 100 km^2 ^should be considered as rather rough estimates despite the fact that they are based on more precise data and might come closer to the true density than achieved in previous studies.

We also demonstrate that the rigorous technique used to identify individuals via a thorough quantitative track survey is a feasible method to study even secretive cats in tropical rainforests. This is the first study that applied this method combined with a capture-recapture model in a study on elusive cats. Earlier studies pointed out some disadvantages of identifying individuals by their tracks [37-39]. Karanth et al. [40] reviewed that 30 years of "pugmark census method [41,42]" to estimate abundances of tigers in India failed because the statistical assumptions for abundance estimates were not considered. In our study, however, we improved the data recording with digital images of the tracks, we enhanced the statistical analysis to separate the individuals and we incorporated the capture probabilities to estimate the abundances. Thus our results show that the method is practicable, as long as research areas are small and inhabited by only a few individuals. This is a major limitation of the track classification method because only a small fraction of the entire population can be distinguished by their tracks. As a rough guideline we would suggest that the study area has to be small enough that less than 10 individuals have to be separated. It also has to be considered that even in small populations (< 10 individuals) two animals may have very similar track measurements and cannot be separated by a PCA. For that reason, the abundance estimates using this method will always result in a minimum abundance. In our study only three track sets clustered with each set having different measurements of right and left front paws. Because this is very unusual, these tracks can almost certainly be assigned to the same individual. We found all good tracks along gravel or former logging roads and on a mud-volcano. For further research this needs to be considered as another limitation of the track classification method because it might be impossible to find enough track sets in densely vegetated habitats. Because clouded leopards like to travel along water courses (pers. obs.), river beds and sand banks along small streams provide a particularly good opportunity to find tracks in forests. Unfortunately, all tracks detected along streams in our study were of poor quality. This might not be the case in other study areas. Although substrate is known to effect the size of tracks profoundly and thus might complicate future studies applying this method, we found greater variation within track sets than between track sets. Therefore, we conclude that substrate did not substantially bias our results.

In our analysis we extrapolated our results to other protected areas, although detailed information on these reserves was lacking. We are aware that this might lead to wrong conclusions in some cases. For example, the consequences of the forest structure (primary or secondary forest) could not be considered in our extrapolation.

The latest faunal survey proved the presence of the species by tracks at one locality only and did not provide any information on population sizes or on the spatial distribution of clouded leopards within the reserves. Therefore our calculations most likely lead to an overestimation. The exclusion of the small reserves and of those reserves from which no data are available, on the other hand, might have led to an underestimate of actual clouded leopard numbers. We want to emphasise that the calculated numbers of clouded leopards in the reserves are very rough estimates and that these numbers can only be a first working hypothesis for future research. Nevertheless, we believe that our calculations are of great value for further management plans because they are the first, however rough, population estimates ever published for clouded leopards and they are based on empirical, although limited, evidence.

Our results show that only four totally protected reserves covering only 5 % of Sabah possess the potential to hold a stable clouded leopard population. However, two of these refuges, Maliau Basin and Danum Valley, cover areas of only 630 km^2 ^and 459 km^2^, hence they might be populated by only 50 to 107 and 37 to 78 individuals, respectively. These numbers lie only slightly above our assumed minimum viable population size.

Since only a small number of animals inhabit totally protected reserves we recommend that higher priority be placed on sustainable management of commercial forest reserves. To ensure the long term persistence of viable clouded leopard populations, the harvest of natural resources from these areas should be limited and controlled. It has to be assumed that clouded leopard densities declined in the recent past as a result of disturbance by logging activities. In contrast to other carnivores such as leopards (*Panthera pardus*) or mountain lions, clouded leopards seem to be less able to adapt to human encroachment. They only rarely prey on domestic animals and avoid habitats around human settlements (unpubl. data). Those behavioural traits lead to the assumption that commercial forest reserves harbour a significant lower density of clouded leopards and that our calculations most probably overestimated their actual numbers. Consequently, those areas have to be larger in order to host viable populations. Illegal hunting of prey animals in some of those areas might also have a negative effect on clouded leopard densities. Direct persecution does not seem to have a substantial negative effect on clouded leopards in Sabah [21]. Further research activities in these harvested areas are needed to reveal to what extent our calculations overestimated the abundances in such commercial forest reserves and what impact logging activities, forest structure and hunting have on the clouded leopard population and its prey.

Compared to other areas where clouded leopards are distributed, Sabah has the potential to protect this species. If the vast commercial forest reserves are protected and sustainably managed, they will serve as refuges for clouded leopards, playing an important role in preventing inbreeding depression by allowing genetic exchange through dispersing animals.

Recent phylogenetic findings suggest that the clouded leopard on Borneo might be a different species (*Neofelis diardi*) [43]. If this proves to be true, research and conservation efforts on behalf of the species are of even greater importance.

## Conclusion

In this paper we present the first population estimates ever published for clouded leopards based on empirical evidence. We demonstrate that the method used by Davies and Payne [26] most likely resulted in an overestimate of the true clouded leopard density. We also show that the technique to identify individuals using a thorough quantitative track survey is a feasible method to study even secretive cats in tropical rainforests. The combination of this kind of track survey with a capture-recapture analysis holds a high potential for further studies if the limitations are well considered. Since our results indicate that only a small number of animals inhabit totally protected reserves we suggest that a higher priority be placed on sustainable management of commercial forest reserves.

Further research will be of great importance in understanding the ecology of clouded leopards in Borneo and their role as top predator within the ecosystem. More surveys are needed throughout Sabah to clarify the status and threats to this elusive species.

## Methods

### Study area

**Figure 5:**
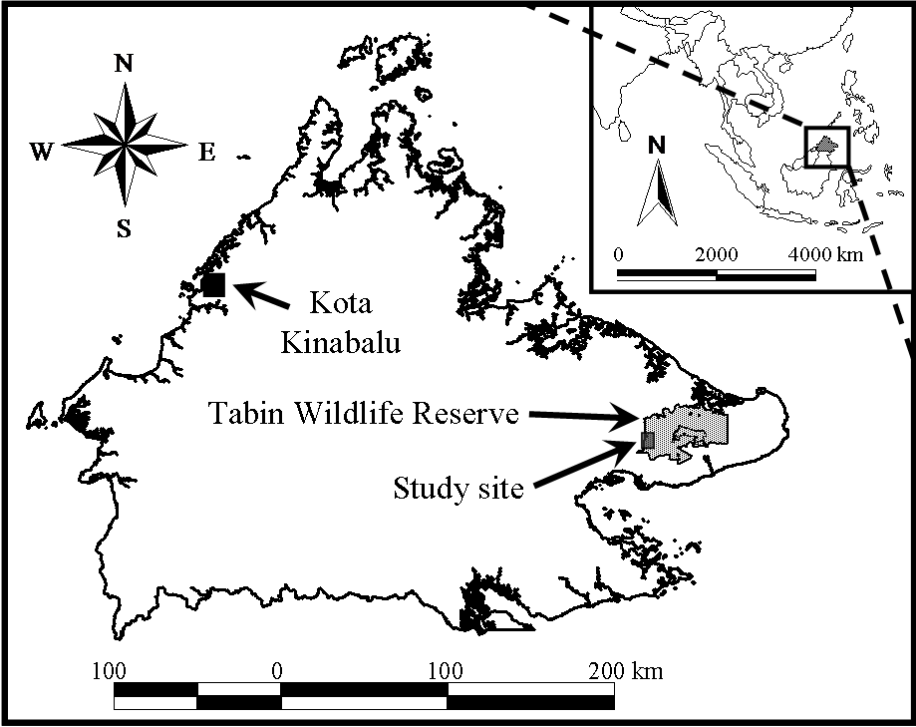
Tabin Wildlife Reserve and the study site in southeast Sabah, Malaysia.

The fieldwork was carried out in Tabin Wildlife Reserve (Tabin or TWR) (5°15'–5°10'N, 118°30'–118°45'E), a 1,205 km^2 ^protected area (gazetted as a Wildlife Reserve in 1984) in the eastern part of the Malaysian State of Sabah in north-eastern Borneo (Fig. 5). Sabah lies close to the equator, possessing a relatively constant tropical climate with an annual rainfall in the study area of about 3000 mm. Tabin is Sabah's largest and oldest wildlife reserve [44,45], currently managed by the Sabah Wildlife Department and the Sabah Forestry Department.

Excluding the so-called core area and seven smaller Virgin Jungle Reserves, all other areas of Tabin (more than 80% of the reserve) have been selectively logged between 1969–1989 [45,46]. No legal logging has taken place after 1989 [47]. Thus, TWR is a mosaic of forest types in different succession stages. A gravel road running north to south along the western boundary separates the forest reserve from the adjacent palm oil (*Elaeis guineensis*) plantations.

The reserve plays an important role as a dedicated ground for the conservation of protected mammals in Sabah. Tabin is home to some endangered flagship mammals, such as the Borneo pygmy elephant (*Elephas maximus borneoensis*), the Sumatran rhinoceros (*Dicerorhinus sumatrensis harrissoni*), the banteng (*Bos javanicus lowi*) and the orang utan (*Pongo pygmaeus pygmaeus*).

The study site was located adjacent to the Tabin field station on the western boundary of the reserve comprising 6 km along the North-South road, and another 6 km east and west along an old logging road.

### Determining the size of the area surveyed

An existing road, trail and stream system was used for all tracking operations. This method promised to be more successful than a square-based area approach with a straight transect grid, because large cats are likely to travel on existing paths [21,48-50]. A buffer was created around each transect to estimate the size of the surveyed area as accurately as possible. To calculate the buffer width, ecological factors of the target species were required [51]. Recent studies used the distance moved by tigers between two photo-recaptures to calculate this parameter [33,39]. However, Soisalo and Cavalcanti [35] recently pointed out that, due to an underestimating of the distance moved by the animals, the calculations might overestimate the true densities. In contrast, other studies used functions of home range size, density and trap spacing to calculate the buffer width [51]. To overcome the uncertainties we considered both approaches and designed the following equation to determine the buffer width *W *in our study:

W=C2+x¯(M)2     (1)
 MathType@MTEF@5@5@+=feaafiart1ev1aaatCvAUfKttLearuWrP9MDH5MBPbIqV92AaeXatLxBI9gBaebbnrfifHhDYfgasaacH8akY=wiFfYdH8Gipec8Eeeu0xXdbba9frFj0=OqFfea0dXdd9vqai=hGuQ8kuc9pgc9s8qqaq=dirpe0xb9q8qiLsFr0=vr0=vr0dc8meaabaqaciaacaGaaeqabaqabeGadaaakeaacqWGxbWvcqGH9aqpdaWcaaqaamaalaaabaWaaOaaaeaacqWGdbWqaSqabaaakeaacqaIYaGmaaGaey4kaSIafmiEaGNbaebacqGGOaakcqWGnbqtcqGGPaqkaeaacqaIYaGmaaGaaCzcaiaaxMaadaqadaqaaiabigdaXaGaayjkaiaawMcaaaaa@3B26@

where *C *is the core area of home range sizes an x¯
 MathType@MTEF@5@5@+=feaafiart1ev1aaatCvAUfKttLearuWrP9MDH5MBPbIqV92AaeXatLxBI9gBaebbnrfifHhDYfgasaacH8akY=wiFfYdH8Gipec8Eeeu0xXdbba9frFj0=OqFfea0dXdd9vqai=hGuQ8kuc9pgc9s8qqaq=dirpe0xb9q8qiLsFr0=vr0=vr0dc8meaabaqaciaacaGaaeqabaqabeGadaaakeaacuWG4baEgaqeaaaa@2E3D@ (*M*) is the average daily movement. Values for *C *(*C *= 6 km^2^) and *M *(*M *= 1.932 km) were obtained from Grassman et al. [9] in Phu Khieo Wildlife Sanctury, Thailand, since there were no data available on these parameters from Borneo. We preferred to use the core area instead of the total home ranges to calculate the size of the area surveyed because long distances travelled by large cats may increase the total home range size significantly.

The North-South road forms the boundary separating Tabin Wildlife Reserve from the adjacent palm oil plantations. A buffer calculated by equation 1 would have overestimated the surveyed area, because it would have included the nearby plantations which do not constitute suitable habitat for clouded leopards. Although clouded leopards were observed entering plantations in Borneo (Sabah Wildlife Department pers. comm.; pers. obs.), presumably following their prey, they were never seen deeper than 300 m inside the palm oil plantation (Sabah Wildlife Department pers. comm.; pers. obs.). Thus it was assumed that a smaller buffer width of 300 m to the west of this road transect would be adequate to describe the survey area.

### Data collection

During March and August 2005, eight transects crossing different habitats were established and each transect was surveyed 20 times. In addition to two transects along the gravel road and one along the old logging road towards the reserve's centre, two transects followed existing jungle trails and three transects followed streams. The total length of all transects was approximately 35 km. Every 250 m a GPS coordinate was taken and a digital map showing all transects was produced using the program ArcGIS 9.1 (ESRI Inc.)

Our sampling unit was a track set (TS), defined as one or more contiguous pugmarks from any paw made by the same clouded leopard. Tracks were photographed with a digital camera (4 mega pixel) fixed on a monopod perpendicular to the track. An umbrella was used to adjust to the light conditions. A scale was placed on two sides of the track to standardize measurements. Only those tracks in good condition with clear edges and in flat terrain were included in the analysis to ensure accurate measuring. GPS coordinates were taken of each track set and later digitized in ArcGIS 9.1 (ESRI Inc.).

### Track measurement

**Figure 6:**
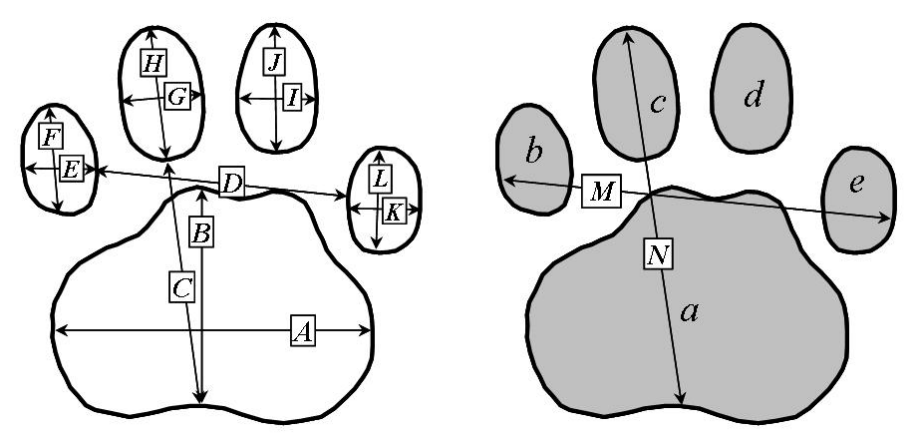
**14 linear and 5 area measurements of the tracks**. A = heel pad width, B = heel pad length, C = heel to second toe length, D = outer toe spread length, E = inner toe width, F = inner toe length, G = second toe width, H = second toe length, I = third toe width, J = third toe length, K = outer toe width, L = outer toe length, M = total width, N = total length, *a *= area of heel pad, *b *= area of inner toe, *c *= area of second toe, *d *= area of third toe, *e *= area of outer toe.

To discriminate individual animals, 14 linear and five area measurements were taken from each track (Fig. 6). Measurement technique were adopted from a variety previous studies [11-13,52] with the intent to increase the level of discrimination. The units of linear and area measurements were millimetres with a 1 mm and 1 mm^2 ^level of precision respectively. Angle measurements, which proved to have a high level of discrimination in previous studies [12,52], varied greatly among tracks of a given individual thus making this tool inadequate for discriminating individuals in our study. All digital track photographs were measured using Adobe Acrobat 7.0 Professional™ (Adobe Systems, Inc.).

### Statistical and analytical analysis

For the analysis, it was presumed that we could differentiate between pugmarks made by front and rear feet as well as by left or right feet. Confusion with pugmarks from other cat species could be excluded as a possibility, because no other large cats are present in Borneo. Confusion with tracks of bay cats (*Catopuma badia*), which might have tracks sizes similar to small clouded leopards, can be ruled out because no confirmed observations of bay cats have been made in Tabin. In order to determine if left and right tracks could be combined for the analysis to enlarge the data set, we used a paired t-test to compare the means of the total width from left and right tracks of each TS. This was done independently for front and rear tracks. We tested the other linear and area measurements as well to determine any differences between the variables. The t-test could be applied because it could be assumed that the means have a normal distribution.

To achieve an optimal separation of each TS, a standardized principal component analysis (PCA) was applied. Principal component (PC) 1 against PC 2 separated individuals better in a scatter plot than two of the original variables did [12]. We excluded the width of the heel pad and of each toe in our analysis, because the information in these variables is correlated highly with the length and area of the heel pads and toes, respectively. The remaining 14 variables were treated as being equally important, having the advantage of coping with linear and area measurements. We favoured the PCA over a discriminant analysis, which has been applied in similar studies [11,13,52], because this method does not require that the number of clouded leopards is known prior to the analysis. The PCA does not classify data into fixed groups of clouded leopards, because the number of groups was unknown. Rather it associates each track with another, even if they derive from the same TS. Tracks from the same TS should cluster together in space, as will tracks from different TSs made by the same individual. All data were analyzed using STATISTICA 6 (StatSoft, Inc. 2001).

After matching the tracks to individual clouded leopards the capture histories for each animal were developed, in a manner utilized by camera-trapping studies [33,36,53-55]. The capture history data were analyzed using the software CAPTURE [29,32,56] developed to implement closed population capture-recapture models. This program uses a number of different models to generate abundance estimates for a sampled area, based on the number of individual animals captured and the frequency of recaptures. The models differ in assumed sources of variation in capture probability, including individual heterogeneity, behavioural response, variation over time and various combinations of these. CAPTURE uses a discriminant function model selection algorithm to provide an objective criterion for selecting the best approximating model. In addition, CAPTURE statistically tests the closure assumption.

The abundance estimates were then used to estimate the clouded leopard densities, defined as *D = N/A*, where *N *is animal abundance and *A *is the effective surveyed area sampled.

### Application of the results on the landscape level

Digital maps of all protected areas within Sabah and the results of their last faunal survey (2000–2001) provided by the Sabah Wildlife Department were used for the large scale analysis. To estimate future prospects of clouded leopards in various protected areas different variables were taken into account. Most important for the evaluation were the presence of clouded leopards, the reserve size, connectivity and classification of the protected areas. We classified the reserves as a) totally protected reserves and b) commercial forest reserves, where the commercial forest reserves are consistent with class 2 of the classification by Sabah Forestry Department. We pooled the classifications of class 5 (mangrove forests) and class 7 (wildlife reserves) within the designation of class 1 (totally protected areas), because in all of these classes hunting and selective logging are prohibited, making them subject to protective conditions. Only areas which were big enough to hold a minimum population of 50 individuals [57,58] were included in the analysis since smaller populations of large cats, such as the Florida panther (*Puma concolor coryi*), experienced reduced viability and fecundity caused by inbreeding [59]. Furthermore, smaller populations are more susceptible to environmental and demographic stochasticity. Therefore a minimum reserve size was calculated based on our density estimation in TWR. Due to a lack of detailed data, we assumed densities to be similar in all protected areas and calculated a rough number of clouded leopards within each reserve, based on the reserve sizes and the density obtained from our results in the TWR.

## Authors' contributions

AW conceived the study, analyzed the data and drafted the manuscript. FF participated in the study design, in the data analysis and in the revision of the manuscript. MAB participated in data collection and helped to coordinate the field work in Malaysia. KEL coordinated the study and participated in its design and in the final revisions. All authors read and approved the final manuscript.

## Figures and Tables

**Table 1:** Estimated abundance of clouded leopards and other relevant statistics for capture-recapture analysis at the research site in Tabin Wildlife Reserve, Sabah, Malaysia

No. of occasions	20
Closure test	0.45
Selection criteria M_0_	1.00
M_h_	0.93
Estimated capture probability per sampling occasion	0.06
Estimated capture probability over all sampling occasions	0.80
No. of animals captured	4
Population estimate ± SE	5 (± 2.26)
Approximately 95% confidence interval	4 to 9
Surveyed area (km^2^)	56
Density (per 100 km^2^)	8.93
Density range using the 95% confidence interval	8 to 17

**Table 2:** Protected areas in Sabah.

Name of protected area	no.	class	size [km^2^]	CL presence	FS	Potential no. of CL
Tabin & Kulamba WR.	1	1	1,409	yes	no	113–240
Danum Valley FR.	2	1	459	yes	no	37–78
Maliau Basin FR.	3	1	630	yes	no	50–107
Tawau Hill & Ulu Kalumpang FR.	4	1	943	yes	no	75–160
Crocker Range NP	5	1	1,406	yes	no	112–239
Kinabatangan WS.	6	1	~450	yes	no	36–77
Ulu Tungud FR.	7	2	1,233	yes	yes	99–210
Trus Madi FR.	8	2	1,759	yes	yes	141–299
Segaluid Lokan FR.	9	2	573	yes	yes	46–97
Deramakot FR.	10	2	551	yes	yes	44–94
Tangkulap FR.	11	2	275	yes	yes	22–47
Malua FR.	12	2	340	yes	yes	27–58
Sg. Pinangah FR.	13	2	2,355	yes	yes	188–400
Gunung Rara FR.	14	2	2,172	yes	yes	174–369
Kalabakan FR.	15	2	2,240	yes	yes	179–381
Pensiangan FR.	16	2	1,031	yes	yes	82–175
Sg. Tagul FR.	17	2	1,058	yes	yes	85–185
Ulu Sg. Milian FR.	18	2	777	unknown	no	unknown
Kuamut FR.	19	2	1,152	unknown	no	unknown
Ulu Segama FR.	20	2	2,013	unknown	no	unknown
Sipitang FR.	21	2	2,589	unknown	no	unknown
Salpulut FR.	22	2	2,419	unknown	no	unknown
Paitan FR.	23	2	711	no	yes	-
Lingkabau FR.	24	2	713	no	yes	-
Mt. Mandalom FR.	25	2	379	no	yes	-
Ulu Sg. Padas FR.	26	2	605	no	yes	-

Total			30,242			1511–3210

CL = clouded leopard, FS = faunal survey, WR = wildlife reserve, FR = forest reserve, WS = wildlife sanctuary, 1 = totally protected area, 2 = commercial forest reserves

## References

[B1] Sillero-Zubiri C, Laurenson MK (2001). Interactions between carnivores and local communities: conflict or co-existence?. Carnivore Conservation.

[B2] Berger J (1999). Anthropogenic extinction of top carnivores andinterspecific animal behaviour: implications of the rapid decoupling of a web involving wolves, bears, moose, and ravens. Proc R Soc Lond B.

[B3] Crooks KR, Soulé ME (1999). Mesopredator release and avifaunal extinctions in a fragmented system. Nature.

[B4] Paine RT (1966). Food web complexity and species diversity. Am Nat.

[B5] Sergio F, Newton I, Marchesi L (2005). Top predators and biodiversity. Nature.

[B6] Terborgh J, Lopez L, Nunez PV, Rao M, Shahabuddin G, Orihuela G (2001). Ecological meltdown in predator-free forest fragments. Science.

[B7] Terborgh J, Feeley K, Silman M, Nunez PV, Balukjian B (2006). Vegetation dynamics of predator-free land-bridge islands. Journal of Ecology.

[B8] Weber W, Rabinowitz AR (1996). A global perspective on large carnivore conservation. Conservation Biology.

[B9] Grassman LI, Tewes ME, Silvy NJ, Kreetiyutanont K (2005). Ecology of three sympatric felids in a mixed evergreen forest in North-Central Thailand. J Mamm.

[B10] Karanth KU, Chundawat RS, Nichols JD, Kumar NS (2004). Estimation of tiger densities in the tropical dry forests of Panna, Central India, using photographic capture-recapture sampling. Animal Conservation.

[B11] Smallwood KS, Fitzhugh EL (1993). A rigorous technique for identifying individual mountain lions Felis concolor by their tracks. Biol Conserv.

[B12] Riordan P (1998). Unsupervised recognition of individual tigers and snow leopards from their footprints. Animal Conservation.

[B13] Grigione MM, Burman P, Bleich VC, Pierce BM (1999). Identifying individual mountain lions Felis concolor by their tracks – refinement of an innovative technique. Biol Conserv.

[B14] Nowell K, Jackson P (1996). Wild cats: Status, Survey and Conservation Action Plan. Gland IUCN.

[B15] Hemmer H (1968). Studien zur Ethologie des Nebelparders Neofelis nebulosa (Griffith 1821) und des Irbis Uncia uncia (Schreber 1775). Veroeffentlichungen der Zoologischen Staatssammlung Muenchen.

[B16] Santiapillai C (1986). The status and conservation of the Clouded leopard (Neofelis nebulosa diardi) in Sumatra. Bogor WWF/IUCN.

[B17] Austin SC, Tewes ME (1999). Ecology of the clouded leopard in Khao Yai National Park, Thailand. Cat News/IUCN SSC.

[B18] Dinerstein E, Mehta JN (1989). The Clouded leopard in Nepal. Oryx.

[B19] Banks E (1931). A popular account of the mammals of borneo. Journal of Malayan Br Roy Asiatic Soc.

[B20] Pocock RI (1939). Family Felidae. Fauna of British India (Mammals).

[B21] Rabinowitz AR, Andau P, Chai PPK (1987). The Clouded leopard in Malaysian Borneo. Oryx.

[B22] Rabinowitz AR (1988). The Clouded leopard in Taiwan. Oryx.

[B23] Ghose D (2002). First sighting of clouded leopard Neofelis nebulosa from the Blue Mountain National Park, Mizoram, India. Current Science (Bangalore).

[B24] Davies RG (1990). Sighting of a clouded leopard (*Neofelis nebulosa*) in a troop of pigrail macaques (*Macaca nemestrina*) in Khao Yai National Park, Thailand. Nat Hist Bull Siam Soc.

[B25] Yamada JK, Durrant BS (1989). Reproductive Parameters of Clouded Leopards (Neofelis nebulosa). Zoo Biology.

[B26] Davies G, Payne J (1982). A faunal survey of Sabah. IUCN/WWF Project No 1692 WWF Malaysia.

[B27] Wood HS (1949). The Clouded Leopard. J Bengal Nat Hist Soc.

[B28] Cat Specialist Group 2002 Neofelis nebulosa. 2006 IUCN Red List of Threatened Species.

[B29] Otis DL, Burnham KP, White GC, Anderson DR (1978). Statistical inference from cature data on closed populations. Wildlife Monographs.

[B30] Jackson R, MacDonald DW (2001). Clouded leopard. The new Encyclopedia of Mammals.

[B31] Trolle M, Kery M (2003). Estimation of ocelot density in the Pantanal using capture-recapture analysis of camera-trapping data. J Mamm.

[B32] White GC, Anderson DR, Burnham KP, Otis DL, Los Alamos (1982). Capture-recapture and Removal MethodsforSampling Closed Populations.

[B33] Karanth KU, Nichols JD (1998). Estimation of tiger densities in India using photographic captures and recaptures. Ecology.

[B34] Nichols JD, Karanth KU, Karanth KU, Nichols JD (2002). Statistical Concepts: Estimating Absolut Densities of Tigers Using Capture-Recapture Sampling. Monitoring Tigers and their Prey.

[B35] Soisalo MK, Cavalcanti SMC (2006). Estimating the density of a jaguar population in the Brazilian Pantanal using camera-traps and capture-recapture sampling in combination with GPS radio-telemetry. Biol Conserv.

[B36] Karanth KU (1995). Estimating tiger populations from camera-trap data using capture-recapture models. Biol Conserv.

[B37] Karanth KU, Tilson RL, Seal US (1987). Tigers in India: A critical review of field cencuses. Tigers of the world.

[B38] Panwar HS (1979). A note on tiger census technique based on pugmark tracings. Indian Forester.

[B39] Kawanishi K (2002). Population status of tigers (Panthera tigris) in a primary rainforest of peninsular Malaysia.

[B40] Karanth KU, Nichols JD, Seidensticker J, Dinerstein E, Smith JLD, McDougal C (2003). Science deficiency in conservation practice: the monitoring of tiger popoulations in India. Animal Conservation.

[B41] Choudhury SR (1970). Let us count our tigers. Cheetal.

[B42] Choudhury SR (1972). Tiger census in India. Cheetal.

[B43] Buckley-Beason VA, Johnson WE, Nash WG, Stanyon R, Menninger JC, Driscoll CA Molecular evidence for species-level distinction in modern clouded leopards (*Neofelis nebulosa*). Current Biology accepted.

[B44] Payne J (1986). Tabin Wildlife Reserve, Sabah: A preliminary managment plan. WWF Malaysia.

[B45] Sale JB (1994). Management Plan for Tabin Wildlife Reserve. Wildlife Dept Ministry of Tourism and Environmental Development Sabah Malaysia Kota Kinabalu Sabah.

[B46] Ecotone Management (1998). Preliminary Environmental Assesments for Upgrading of 23 km Forest Road in Tabin Wildlife Reserve, Lahad Datu, Sabah. Ecotone Management.

[B47] Malim TP, Mohamed M, Mohamed M, Andau M, Dalimin MN, MTP (1999). Butterfly Monitoring using Point Count Method at Tabin Wildlife Reserve, Lahad Datu, Sabah. Tabin Scientific Expedition.

[B48] Mills MGL, van Heerden J (1997). Census techniques for large carnivores. Proceedings of a Symposium on Lions and Leopards as Game Ranch Animals.

[B49] Wilson GJ, Delahay RJ (2001). A review of methods to estimate the abundance of terrestrial carnivores using field signs and observations. Wildlife Research.

[B50] Henschel P, Ray J (2003). Leopards in African rainforests: Survey and monitoring techniques. WCS Global Carnivore Program.

[B51] Wilson KR, Anderson DR (1985). Evaluation of two density estimators of small mammal population size. J Mamm.

[B52] Lewison R, Fitzhugh EL, Galentine SP (2001). Validation of a rigorous track classification technique: identifying individual mountain lions. Biol Conserv.

[B53] Silver SC, Ostro LET, Marsh LK, Maffei L, Noss AJ, Kelly MJ (2004). The use of camera traps for estimating jaguar Panthera onca abundance and density using capture/recapture analysis. Oryx.

[B54] Kawanishi K, Sunquist ME (2004). Conservation status of tigers in a primary rainforest of Peninsular Malaysia. Biol Conserv.

[B55] Karanth KU, Nichols JD, Kumar NS, Thompson WL (2004). Photographic Sampling of Elusive Mammals in Tropical Forests. Sampling Rare or Elusive Species.

[B56] Rexstad E, Burnham KP (1991). User's Guide for Interactive Program CAPTURE Abundance Estimation of Closed Populations.

[B57] Shaffer ML (1981). Minimum population sizes for species conservation. BioScience.

[B58] Allen CR, Pearlstine LG, Kitchens WM (2001). Modeling viable mammal populations in gab analysis. Biol Conserv.

[B59] Roelke ME, Martenson JS, O'Brien SJ (1993). The consequences of demographic reduction and genetic depletion in the endangered Florida pather. Current Biology.

